# The Influence of Wound Closure Techniques after Surgical Decompression in Patients with Carpal Tunnel Syndrome on Sleep Disturbance and Life Quality: A Prospective Comparison of Surgical Techniques

**DOI:** 10.3390/clinpract14020042

**Published:** 2024-03-26

**Authors:** Veridijana Sunjic Roguljic, Luka Roguljic, Ivana Jukic, Vedran Kovacic

**Affiliations:** 1 Plastic, Reconstructive and Aesthetic Surgery with Burn Care Division, Surgery Department, University Hospital of Split, 21000 Split, Croatia; lroguljic@kbsplit.hr; 2 Orthopaedics and Traumatology Division, Surgery Department, University Hospital of Split, 21000 Split, Croatia; 3 Gastroenterology Division, Internal Medicine Department, University Hospital of Split, 21000 Split, Croatia; 4 University Department of Health Studies, University of Split, 21000 Split, Croatia; 5 Division of Emergency and Intensive Medicine with Clinical Pharmacology and Toxicology, Internal Medicine Department, University Hospital of Split, 21000 Split, Croatia; 6 School of Medicine, University of Split, 21000 Split, Croatia

**Keywords:** insomnia, sleep disturbance, life quality, carpal tunnel, skin adhesive, skin suture, cyanoacrylate

## Abstract

Background: The compression of the median nerve within the carpal tunnel is the cause of carpal tunnel syndrome (CTS). Surgical decompression is successful in improving sleep and quality of life, but the effect of tissue adhesives as a material for wound closure has not been investigated. The objective of the study was to evaluate sleep disorders and health-related life quality by comparing two methods for wound closure after carpal surgery in participants who were randomized to receive tissue adhesives or transcutaneous sutures. Methods: The subjects, aged 61.56 ± 12.03 years, were randomized to receive either tissue adhesives (*n* = 50) or suture-based wound closure (*n* = 50) using the Glubran Tiss 2^®^ skin adhesive after subcutaneous running sutures. The outcomes were assessed during the 12-month postoperative follow-up. The Pittsburgh Sleep Quality Index (PQSI) and Insomnia Severity Scale (ISI) were used for the sleep disturbance assessment, and for the health-related quality of life assessment, the total SF-36 (36-Item Short Form Survey) was used. Results: The PQSI, ISI, and SF-36 were not statistically different between groups during the follow-up, except in the ISI score two weeks after surgery (9.40 ± 1.18 in the tissue adhesive group vs. 9.96 ± 1.09 in the suture-based group, *p* = 0.008). The PQSI, ISI, and SF-36 scores for all the subjects and groups were persistently improved at all the follow-up intervals after surgery. The total SF-36 score increased 12 months after surgery (49.84 ± 5.85 vs. 82.46 ± 5.68, *p* < 0.001). Conclusions: Cyanoacrylate-based adhesion material can be used for wound closure after open CTS decompression as a standard transcutaneous suture, and both techniques equally lead to improved sleep and life quality. The possible advantages of tissue adhesives include a faster reduction in the ISI.

## 1. Introduction

The most prevalent entrapment neuropathy affecting the upper limb is carpal tunnel syndrome (CTS), which frequently requires carpal tunnel release (CTR) for decompression in cases where conservative treatment is ineffective [1]. In the distribution of the median nerve, patients with CTS frequently suffer with pain, tingling, and numbness. These symptoms frequently interfere with sleep, which can have a significant negative impact on a patient’s quality of life [2].

It has been proven that surgical decompression of the carpal tunnel is successful in improving the sleep and quality of life of patients with CTS [3]. Even non-surgical methods such as steroid injection treatment or medicaments have a significant effect on sleep improvement in patients with CTS [4,5]. It is of the utmost importance for the patient to achieve relief of night paresthesia and improved quality of sleep by treating CTS, which then has an impact on their quality of life and ability to work [6].

Differences in the approach to CTS treatment and the advantages of one procedure over another have been analyzed in various clinical studies, while different clinical parameters have been used as the outcome measures, most often patient-reported questionnaires, physical examination of scars, medial nerve conduction testing, or the number of surgical complications [7].

Despite plenty of surgical procedures and adjustments that have been proposed to improve the neurological, functional, and aesthetic outcomes of CTS decompression [8], to date, there has been no comparison analysis of different surgical methods aimed at enhancing sleep and life quality in CTS patients.

Tissue adhesives, including 2-octylcyanoacrylate, have been recently used in clinical settings to seal wounds following surgery since they have a high tensile strength and bacteriostatic properties [9]. Despite several proposed wound closure procedures, there is a remarkable paucity of data on the potential relevance of tissue adhesives to open carpal tunnel decompression surgery. In the small number of studies comparing tissue adhesives and sutures for wound closure after carpal surgery, the observed outcomes were mostly aesthetic [10]. The effect of using tissue adhesives as the material for wound closure during CTS decompression on sleep disturbances and health-related life quality has not been studied.

The objective of the current study was to evaluate sleep disorders and health-related life quality by comparing two methods for wound closure after carpal surgery in participants who were randomized to receive tissue adhesives or transcutaneous sutures as the wound closure material. Therefore, this study is the first to aim to find the impact of different wound closure techniques after carpal tunnel decompression on sleep disorders (measured using the Insomnia Severity Scale and the Pittsburgh Sleep Quality Index) and health-related life quality (measured using the Short-Form Health Survey). We hypothesized that various wound closure techniques have a significantly different impact on life and sleep quality assessments.

## 2. Materials and Methods

A randomized prospective follow-up single-center trial was carried out at the Surgery Department of the University Hospital of Split. The Ethics Committee at the hospital approved the study with an ethics code of 500-03/22-01/41 and a date of approval of 31 March 2022. The Helsinki Declaration’s guiding principles were followed in the conduct of the study, as were the Consolidated Standards of Reporting Trials (CONSORT) guidelines. Informed consent was obtained from all the subjects involved in the study. This study was part of a clinical project registered with www.clinicaltrials.gov (accessed on 12 April 2023) (NCT05808855). The participants in the study included adult patients (>18 years) with carpal tunnel syndrome who had failed conservative therapy for more than 6 months and had serious limitations, such as severe median nerve conduction impairment as determined using electromyography, thenar atrophy, or thumb abduction weakness. Median nerve conduction studies were performed in all the subjects to assess the conduction of the median nerve through the carpal tunnel (speed, latency, and amplitude of motor and sensory conduction). All the included subjects had at least one of the following motoric or sensory median nerve impairments: a sensory conduction velocity > 45 m/s, a peak distal sensory latency < 3.5 ms, a sensory nerve action potential amplitude > 10 mV, a distal motor latency < 4.2 ms, a distal motor amplitude > 5 mV, and a motor conduction velocity > 50 m/s. The exclusion criteria included a history of severe general illness with cachexia, a family history of keloids or hypertrophic scars, a history of previous wrist trauma or surgery, another etiology of neuropathy, and threatening hemorrhagic complications (patients with peroral anticoagulation and/or antithrombotic therapy).

The sample size was calculated taking into account the expected variability in the main outcome measure (SF-36) from previous studies with an alpha error of 0.05 to detect a significant difference and an 80% test power (beta error of 0.2). For this power of the study, the minimum number of subjects was estimated to be 37 in each group.

**Figure 1:**
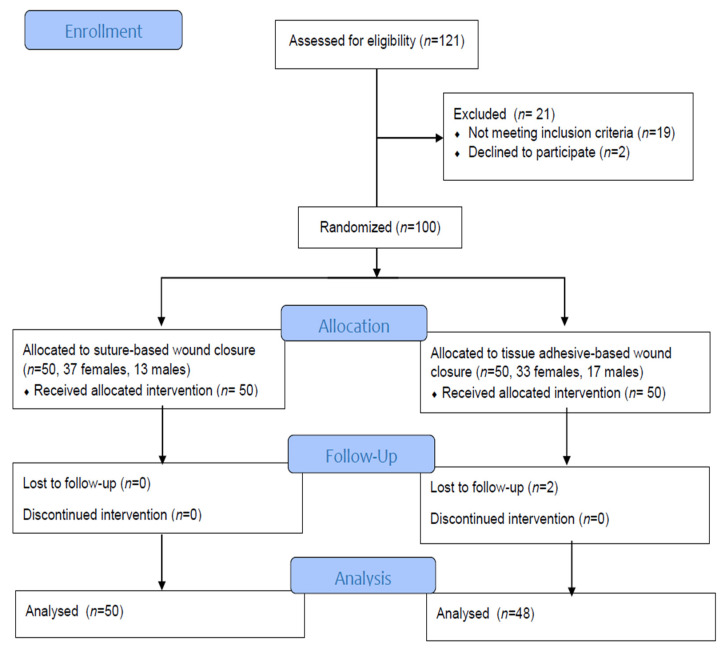
Study flow diagram (CONSORT).

After applying the inclusion criteria, 100 patients were included in the trial. During the 12-month postoperative follow-up period, two subjects dropped out. A total of 98 subjects were included (Figure 1). Subjects were randomized to receive either tissue-adhesive-based or suture-based wound closure. A computer created the randomization, which consisted of numbers at a 1:1 ratio between the two interventions.

A total of 6 licensed plastic surgeons in the Plastic, Reconstructive and Aesthetic Surgery with Burn Care Division of the Surgery Department of the University Hospital of Split, Croatia, participated in the surgical procedures. All the surgical procedures were performed as standard open carpal canal decompression surgery [11]. After preparation of the operating field with a tourniquet and local infiltration of 2% lidocaine into the soft tissue of the palm and carpal tunnel, a 15–18 mm long skin incision was made following the radial half of the palm, not over the joint flexor. To open the skin and transect the carpal ligament in the proximal direction, scalpel no. 15 was used, and Metzenbaum scissors were used to cut the carpal ligament in the distal direction. Depending on the subject’s randomization group, two different techniques were used for the primary wound closure:

(1) Transcutaneous nylon sutures (non-absorbable polypropylene/polyethylene monofilament, 3/8 needle, thread size 4/0) were used to stitch the skin (Optilene^®^, B. Braun Surgical, S.A., Barcelona, Spain)

(2) After a subcutaneous buried running continuous stitch (thread size 4-0 coated Vicryl^TM^ Plus PS-2, 3/8, Ethicon Inc., Bridgewater, MA, USA), a synthetic two-component surgical glue, Glubran Tiss 2^®^ (GEM S.r.l., Viareggio, Italy), was applied. For the wound closure, 0.35 mL of Glubran Tiss 2^®^ was used and rested for 20 s in the open wound to allow the polymerization process to begin before bandaging. Glubran Tiss 2^®^ is a synthetic bio-inert surgical glue composed of n-butyl 2-cyanoacrylate (NBCA) and 2-octyl cyanoacrylate (OCA) with hemostatic, bacteriostatic, and sealing properties. When applied to wet tissue, Glubran Tiss 2^®^ rapidly polymerizes into a thin, elastic film with a remarkable tensile strength, clinging tightly to the structure of the tissue [12].

All the subjects had the same postoperative care. The postoperative care consisted of the application of a compression bandage for one day and the administration of analgesics. Some subjects needed drainage with a narrow plastic tube on the first two postoperative days. Regular visits by surgeons and nurses with wound dressings were performed on a daily basis. We did not use a splint to immobilize the limb after surgery, but all subjects had to rest strictly to prevent wound dehiscence.

For each subject included in the study, their gender, weight, height, and previous illnesses were recorded before surgery. Subjects were evaluated 2 weeks after surgery for a standard follow-up examination that included wound inspection and suture removal. The next evaluation was performed 6 weeks after surgery, the third evaluation was performed 24 weeks after surgery, and the final evaluation was performed 12 months after surgery.

At the beginning of the study, sleep quality and health-related quality of life were assessed on self-reported scales. For the sleep quality assessment, two validated scales were used: the Pittsburgh Sleep Quality Index (PQSI) and the Insomnia Severity Scale (ISI).

The PSQI is a self-reported questionnaire that assesses the overall sleep quality across 19 items belonging to one of seven different subcategories: subjective sleep quality, sleep latency, sleep duration, habitual sleep efficiency, sleep disturbances, use of sleeping medication, and daytime dysfunction. The scale provides an overall score ranging from 0 to 21, with lower scores indicating a better sleep quality [13].

The ISI was developed to detect and quantify patients’ perception of the severity of insomnia, as well as to assess its impact on daytime functioning and track the treatment response [14]. The ISI self-reported questionnaire consists of seven questions that are added together to yield a total score: 0–7 = no clinically significant insomnia; 8–14 = subthreshold insomnia; 15–21 = clinical insomnia (moderate severity): 22–28 = clinical insomnia (severe).

The PQSI and ISI were evaluated at the beginning of the study (immediately before surgery) and during follow-up visits at 2, 6, and 24 weeks.

The TSF-36 (36-Item Short-Form Survey) is a self-reported measure of health-related quality of life. The SF-36 Health Survey was developed at RAND Health Care as part of the Medical Outcomes Study [15]. It comprises 36 questions that consider eight different dimensions of health (physical and mental components): limitations in physical activities due to health problems, limitations in social activities due to physical or emotional problems, limitations in usual role activities due to physical health problems, bodily pain, general mental health, limitations in usual activities due to emotional distress, vitality, and general health perceptions. The scores are converted into a scale with a minimum of 0 (the worst condition) and a maximum of 100 (the best condition).

The total SF-36 score was calculated at the beginning of the study (immediately before surgery) and at the last follow-up visit at 12 months.

The distributions of the quantitative variables were checked for normality using the Kolmogorov–Smirnov test. Fisher’s exact chi-square test was used to compare the qualitative data between groups. A paired and an unpaired Student’s t-test were used to compare the quantitative data. The statistical analysis was carried out using the Windows version of the SPSS program (IBM SPSS Statistics for Windows, version 26.0, Armonk, NY, USA). *p*-values less than 0.05 were regarded significant.

## 3. Results

The study population comprised 100 patients (30 males and 70 females) who were randomized 1:1 to receive either glue-based wound closure (*n* = 50) or suture-based wound closure (*n* = 50). Two participants dropped out after postoperative follow-up. A total of 98 subjects were evaluated. The age of the study population cohort was 61.56 ± 12.03 years. Right-side surgery was performed on 57 subjects, while left-side surgery was performed on 43 subjects.

The initial parameters and output measures before surgery are demonstrated in Table 1. There were no statistical differences between the groups.

The differences between the two groups in the outcome measures during follow-up are demonstrated in Table 2. We demonstrated a statistically significant reduction in the ISI score (Insomnia Severity Scale) two weeks after surgery in patients whose surgical wounds were closed with tissue adhesives. Some of the other output measures of sleep- and health-related quality of life tended to be better in the group where the surgical wound was closed with glue, but statistical significance was not reached.

**Figure 2:**
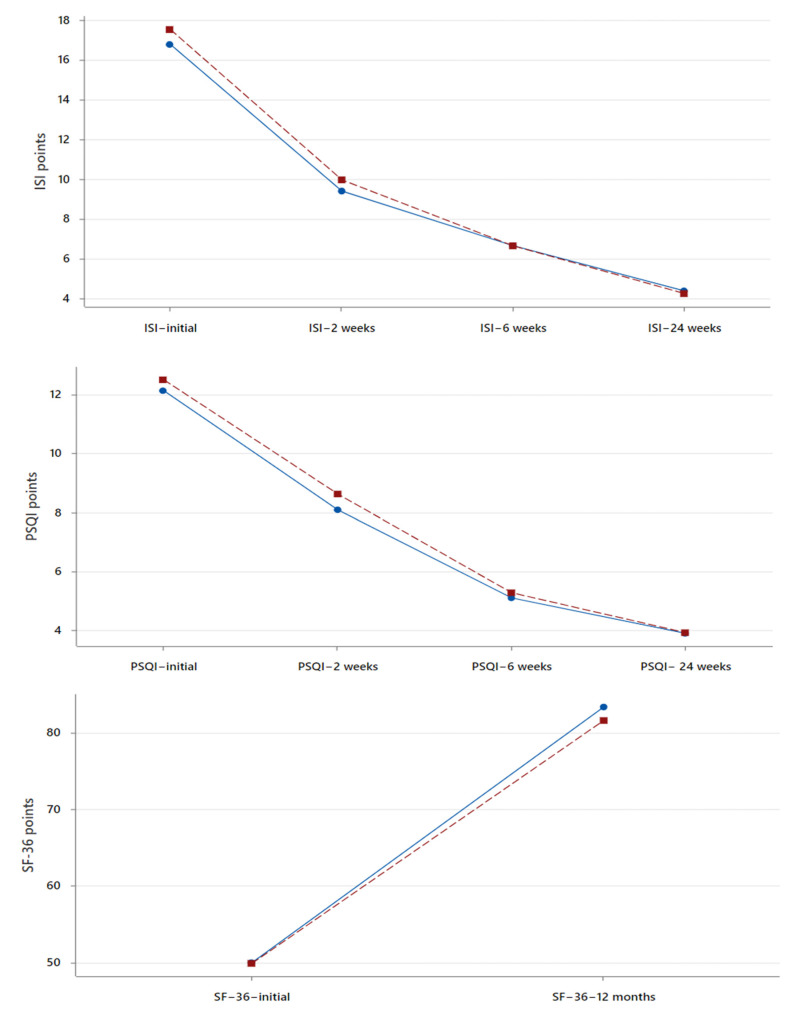
Plots of interval changes in mean values of Insomnia Severity Scale (ISI), Pittsburgh Sleep Quality Index (PQSI), and Short-Form Health Survey (SF-36) over the study duration. The Blue line indicates glue based, and the Red line indicates suture based.

And finally, we demonstrated a significant improvement in the sleep- and health-related quality of life between all the follow-up intervals as a consequence of the continuous recovery of quality of life after surgery. This effect was proven for all subjects (Table 3) and for each group of subjects separately (Figure 2).

## 4. Discussion

The results of this study demonstrated for the first time the impact of using tissue adhesive to close postoperative wounds after the decompression of the carpal tunnel on sleep disorders and quality of life indicators. The study compared standard transcutaneous sutures with 2-octylcyanoacrylate-based tissue adhesive and showed that both techniques of wound closure after carpal tunnel surgery have an equally significant effect on the improvement of sleep disturbances and quality of life. This beneficial effect was significant and long-lasting, with a tendency for constant improvement during follow-up.

A limited number of studies have been conducted to compare different treatment techniques for CTS and their impact on sleep quality and sleep-related quality of life. Although it has been proven that quality of sleep and quality of life improve significantly after surgical decompression of the carpal tunnel [16], there are only a small number of studies conducted as comparative trials between surgical decompression and other treatment methods regarding sleep disturbance and its impacts on life quality. For example, one analysis compared open and endoscopic carpal tunnel release for treating secondary sleep symptoms due to CTS, and it was shown that the endoscopic method leads to a faster resolution of sleep problems compared to the open technique [17].

Improving the quality of sleep is a very important outcome of the treatment of patients with CTS since a patient’s quality of life is greatly impacted by sleep disturbances. About 80% of CTS patients demonstrated clinically significant sleep disturbances (a PSQI score > 5) [18]. The beneficial effect of surgical decompression of the carpal tunnel on sleep disorders has been proven in several studies, and this effect is considered a very important outcome of the surgical treatment of patients with CTS, as sleep improvements have an impact on patient satisfaction and quality of life. Similar to our results, Niedermeier et al. [19] reported an improvement in sleep disorders measured using the Pittsburgh Sleep Quality Index, from a mean of 10.4 points preoperatively to 7.8 points two weeks after surgery and 6.4 points six weeks after surgery. Sleep improvement typically occurs very quickly after surgery. Tulipan et al. [20], in a prospective study, showed an enhancement in the quality of sleep measured as an ISI score within 7 days after CTS open decompression. The authors reported that the ISI score did not further significantly improve in the follow-up period between 2 weeks and 3 months. On the contrary, in our study, we demonstrated a progressive and significant improvement in sleep quality during all 24 weeks of the monitoring period in both groups of subjects. Nevertheless, the favorable effects of carpal tunnel surgery on sleep parameters are long-lasting; for example, Okkesim et al. [21] showed that in the third to sixth month after operative median nerve decompression, individuals with carpal tunnel syndrome had better sleep metrics and a better quality of sleep. Our results are consistent with previous reports, and we demonstrated that an improvement in sleep has a long-term effect with a significant and progressive improvement during the follow-up period. The same effect was achieved in the application of both surgical techniques, with a significantly different improvement in the ISI score 2 weeks postoperatively in subjects with tissue-adhesive-based wound closure compared with the suture-based wound closure group. This may indicate that the sleep improvement effect was achieved faster in the tissue adhesive group. It should be noted that the two surgical approaches used to close the surgical wound—one using transcutaneous sutures and the other using glue plus a subcutaneous running continuous stitch—caused these notable postoperative differences in earlier sleep improvements. The observed notable variations in the outcome measures after surgery can be ascribed not only to the use of tissue glue but also to the distinction between subcutaneous and transcutaneous sutures. In particular, transcutaneous suturing covers a greater surface area of the sutured tissue (skin and subcutaneous tissue). In contrast, the tension of the wound is evenly distributed with less pressure from the sutures on the skin where the nociceptors are located when a subcutaneous suture using tissue glue is used. As a result, pain and discomfort are minimized, which leads to better sleep.

A rapid improvement in symptoms can be important in patients who have very pronounced sleep disorders. In contrast, some authors showed very late effects on improving sleep after surgical decompression of the carpal tunnel; for example, Trouw et al. [22] reported a measurable and significant improvement in the PSQI score at the 12- to 24-month follow-up.

One frequently utilized tool in orthopedic studies is the 36-item Short-Form Health Survey (SF-36) because it is a valid instrument for evaluating health-related quality of life [23]. Nevertheless, the SF-36 has been sparingly used in CTS patients, where it was shown that patients with CTS have a lower SF-36 score compared to the general population, with a significant increase in the SF-36 score after operative decompression of the carpal tunnel [24]. Similarly, our research clearly showed a significant improvement in the total SF-36 score from 49.84 ± 5.85 at the beginning to 82.46 ± 5.68 at the end of the study, 12 months after the surgical decompression. A significant improvement in the SF-36 score was recorded in both groups of subjects. Chen et al. [25] reported a study conducted on 49 CTS subjects treated using nasal instruments and the mini-incision approach as a modification of the standard surgical procedure, which demonstrated a significant increment during the mean follow-up of 13 months in all the SF-36 subdivisions: the mean SF-36 scores for social functioning were 60.3 preoperatively vs. 79.8 postoperatively, and the mean SF-36 scores for mental health were 46.5 preoperatively vs. 65.8 postoperatively. Galasso et al. [26] demonstrated in 30 CTS subjects referred for standard surgery a significant improvement in the mean SF-36 total score from 38.7 to 44.3 points six months after surgery.

Although we hypothesized that different surgical wound closure techniques would have an impact on improvements in sleep and quality of life, we did not prove major differences, except for a short-term improvement in the Insomnia Severity Scale two weeks postoperatively in favor of the group of patients whose wound was closed with tissue glue. Furthermore, in the other outcome measures during the postoperative follow-up, there was a tendency towards better outcomes in the group whose surgical wounds were closed with tissue adhesive, but the statistical significance threshold was not met by those differences. The above may indicate, however, some advantages of tissue adhesives in closing post-surgical wounds during open decompression of the carpal tunnel; however, these differences are not significant from a clinical standpoint. The utility of tissue adhesives for wound closure after CTS decompression was insufficiently studied and was limited to aesthetic outcomes [27]. This is the first study to prove the effectiveness and non-inferiority of using a tissue adhesive to close surgical wounds during open carpal tunnel surgery to improve quality of sleep and life.

However, the study presented here has some limitations. First, this trial was restricted to a single center. Second, the follow-up period is insufficient to draw a conclusion about the intervention’s long-term effects. Third, additional sleep and life quality parameters have to be monitored to better understand the impact of the intervention.

## 5. Conclusions

The study demonstrated that cyanoacrylate-based adhesion material can be used for wound closure after open CTS decompression to the same extent as standard surgical wound closure with transcutaneous sutures, and both techniques lead to a significantly improved sleep quality and health-related life quality. The possible advantages of tissue adhesive over sutures include a faster reduction in the Insomnia Severity Scale. To clarify the findings of this study, larger multicentric trials with a longer follow-up and additional outcomes are needed.

## Figures and Tables

**Table 1:** The initial preoperative differences in characteristics and outcome measures between two groups of participants (glue-based wound closure and suture-based wound closure), mean ± standard deviation or number of participants and percent, Student’s *t*-test for independent samples, and Fisher’s exact chi-square test, one-tailed.

	Glue-Based Technique	Suture-Based	
	*N =* 48	*N =* 50	*p*
Age (years)	63.02 ± 12.97	60.10 ± 10.95	0.113
Body mass index (kg/m^2^)	24.79 ± 3.17	25.04 ± 2.25	0.325
Female sex (*n*, %)	33 (68.75%)	37 (74.00%)	0.257
ISI, initial	16.77 ± 3.05	17.52 ± 3.08	0.115
PSQI, initial	12.15 ± 1.61	12.52 ± 1.67	0.131
SF-36, initial	49.96 ± 5.96	49.86 ± 5.83	0.467

Legend: ISI: Insomnia Severity Scale, PQSI: Pittsburgh Sleep Quality Index, SF-36: Short-Form Health Survey.

**Table 2:** The differences in postoperative outcome measures between two groups of participants (glue-based wound closure and suture-based wound closure), mean ± standard deviation, Student’s *t*-test for independent samples, one-tailed.

	Glue-Based Technique	Suture-Based	
	*N =* 48	*N =* 50	*p*
ISI, 2 weeks	9.40 ± 1.18	9.96 ± 1.09	0.008 *
ISI, 6 weeks	6.65 ± 1.25	6.64 ± 1.10	0.490
ISI, 24 weeks	4.38 ± 1.16	4.24 ± 0.96	0.265
PSQI, 2 weeks	8.08 ± 1.77	8.62 ± 1.59	0.059
PSQI, 6 weeks	5.08 ± 1.01	5.26 ± 1.29	0.227
PSQI, 24 weeks	3.88 ± 1.04	3.90 ± 0.93	0.450
SF-36, 12 months	83.35 ± 4.81	81.59 ± 6.36	0.064

Legend: ISI: Insomnia Severity Scale, PQSI: Pittsburgh Sleep Quality Index, SF-36: Short-Form Health Survey, * *p* < 0.05.

**Table 3:** The follow-up interval differences in outcome measures for all participants (*N =* 98), Student’s *t*-test for paired samples, one-tailed.

	Mean ± Std. Deviation	Mean ± Std. Deviation	*p*
ISI, initial vs. ISI, 2 weeks	17.15 ± 3.07	9.68 ± 1.16	<0.001 *
ISI, 2 weeks vs. ISI, 6 weeks	9.68 ± 1.16	6.64 ± 1.17	<0.001 *
ISI, 6 weeks vs. ISI, 24 weeks	6.64 ± 1.17	4.31 ± 1.06	<0.001 *
PSQI, initial vs. PSQI, 2 weeks	12.34 ± 1.64	8.36 ± 1.69	<0.001 *
PSQI, 2 weeks vs. PSQI, 6 weeks	8.36 ± 1.69	5.17 ± 1.16	<0.001 *
PSQI, 2 weeks vs. PSQI, 24 weeks	5.17 ± 1.16	3.89 ± 0.98	<0.001 *
SF-36, initial vs. SF-36, 12 months	49.84 ± 5.85	82.46 ± 5.68	<0.001 *

Legend: ISI: Insomnia Severity Scale, PQSI: Pittsburgh Sleep Quality Index, SF-36: Short-Form Health Survey, * *p* < 0.05.

## Data Availability

The data presented in this study are available on request from the corresponding author.
